# Potential mechanisms of hemorrhagic stroke in elderly COVID-19 patients

**DOI:** 10.18632/aging.103335

**Published:** 2020-06-11

**Authors:** Haili Wang, Xiaojia Tang, Hongyang Fan, Yuhan Luo, Yuxia Song, Yao Xu, Yingzhu Chen

**Affiliations:** 1 Department of Neurology, Clinical Medical College, Yangzhou University, Yangzhou 225000, Jiangsu, China; 2 Department of Neurology, Clinical Medical College of Yangzhou, Dalian Medical University, Yangzhou 225000, Jiangsu, China

**Keywords:** COVID-19, SARS-CoV-2, hemorrhagic stroke, ACE2, immunity

## Abstract

The novel severe acute respiratory syndrome coronavirus 2 is the causative agent of coronavirus disease 2019, a new human infectious disease. While fever, cough, and respiratory distress are typical first symptoms, a fraction of those affected present instead with neurological symptoms suggestive of central nervous system compromise. This review summarizes the potential contribution of coronavirus disease 2019 to hemorrhagic stroke in the elderly and proposes possible mechanisms. Reports show that the most affected patients have underlying chronic diseases such as hypertension and diabetes, which are two key risk factors for hemorrhagic stroke. Angiotensin-converting enzyme 2 is the main host cell surface receptor interacting with the severe acute respiratory syndrome coronavirus 2 spike glycoprotein to allow viral entry and infection. We speculate that ensuing downregulation of angiotensin-converting enzyme 2 expression may compound the risk conferred by pre-existing comorbidities and critically influence the pathogenesis of hemorrhagic stroke by elevating blood pressure and impairing cerebrovascular endothelial function. Additionally, both age- and/or disease-related immune dysfunction and enhanced catecholamine release secondary to anxiety and stress may also aggravate central nervous system symptoms of severe acute respiratory syndrome coronavirus 2 infection. Thus, assessment of systemic inflammatory biomarkers and tight control of hemodynamic parameters upon admission are crucial to minimize mortality and morbidity in coronavirus disease 2019 patients with central nervous system symptoms suggestive of incipient stroke.

## INTRODUCTION

Since the initial report of cases in Wuhan, Hubei Province, China, in December 2019 and January 2020, coronavirus disease 2019 (COVID-19) has been recognized as a new human disease [1]. The causative agent was identified as a novel coronavirus strain, named severe acute respiratory syndrome coronavirus 2 (SARS-CoV-2) by the Coronavirus Study Group (CSG) [2]. The mortality rate of SARS-CoV-2 is lower than those of Middle East respiratory syndrome coronavirus (MERS-CoV) and severe acute respiratory syndrome coronavirus (SARS-CoV) [3]. However, SARS-CoV-2 spreads more rapidly than MERS-CoV and SARS-CoV because viral load and infectiousness peak before or around the time of symptom onset, i.e. much earlier than for both MERS-CoV and SARS-CoV [3]. The high transmissibility of SARS-CoV-2 is denoted by a basic reproduction number (R_0_) of 3.39 over the whole epidemic period [4]. Moreover, COVID-19 can be transmitted by asymptomatic carriers during the incubation period [4–7], probably because they carry viral loads similar to those of symptomatic patients [8]. Although further studies are warranted to ascertain the epidemiological significance of the asymptomatic cases, this suggests that asymptomatic transmission may be playing a substantial role in the outbreak [6, 9]. Notably, it is increasingly apparent that in many patients, neurological signs and symptoms are the first manifestations of COVID-19 infection [10, 11]. Although clinical data is not enough, there is still much concern that COVID-19 may increase the risk or trigger the onset of hemorrhagic stroke, especially in older patients. This review summarizes common risk factors for both stroke and COVID-19 severity, and potential mechanisms influencing the onset of hemorrhagic stroke in the elderly.

### Identification of SARS-CoV-2 as the causative agent of COVID-19

Zhou et al. provided the first evidence that COVID-19 is associated with a novel coronavirus strain [12]. They used next-generation sequencing and pan-CoV Polymerase Chain Reaction (PCR) primers to determine the cause of the disease in 7 patients with COVID-19 in Hubei, most of whom were seafood market sellers or deliverers [12]. Their findings significantly strengthened the etiological association reported by investigators from India [13], Switzerland [14] and other places in China [15], who had also isolated the novel coronavirus from patients with COVID-19. These efforts, corroborated by statements from Chinese authorities, conclusively led to identification of SARS-CoV-2 as the causative agent of the COVID-19 outbreak [14].

Since its discovery, the sequence of the complete genome of SARS-CoV-2 has been determined [13, 16, 17]. It has ~29,000 nucleotides in length and like other CoVs, it contains at least six open reading frames (ORFs) and several accessory genes [13]. According to Chen et al. [15], the genome sequence of SARS-CoV-2 is 89% identical to the bat SARS-like-CoVZXC21 and 82% identical to the human SARS-CoV [15]. In addition, phylogenetic analysis indicated that two bat SARS-Like CoVs were the nearest homologs of SARS-CoV-2 [13]. Based on genomic structure and phylogenetic analysis, the subfamily Coronavirinae are divided into four genera, namely Alphacoronavirus, Betacoronavirus, Gammacoronavirus, and Deltacoronavirus [13, 18, 19]. Currently, seven human CoVs have been reported: 229E (HCoV-229E), OC43 (HCoV-OC43), NL63 (HCoV-NL63), HKU1 (HCoV-HKU1), SARS-CoV, MERS-CoV, and SARS-CoV-2. HCoV-229E and HCoV-NL63 belong to the Alphacoronavirus genus, while HCoV-HKU1, SARS-CoV, MERS-CoV, and HCoV-OC43 are Betacoronavirus members [18]. SARS-CoV-2 is also classified as a novel Betacoronavirus belonging to the subgenus Sarbecovirus of the Coronaviridae family [13, 15].

The 3’ terminal one-third of SARS-CoV-2 genome sequence encodes four structural proteins, namely spike protein (S), envelope protein (E), membrane protein (M), and nucleocapsid protein (N). Among these, the S gene is particularly important for receptor binding and host specificity [13]. Infection by CoV begins with the binding of the S protein, a surface antigen determining viral tropism, to cell-surface molecules expressed in host cells [20]. As shown in Table 1, host receptors for the seven human CoVs include human aminopeptidase N (CD13) for HCoV-229E [21]; 9-O-acetylated sialic acid for HCoV-OC43 [22]; angiotensin-converting enzyme 2 (ACE2) for SARS-CoV [22]; ACE2 for HCoV-NL63 [23, 24]; 9-O-acetylated sialic acid for HCoV-HKU1 [25, 26]; dipeptidyl peptidase 4 (DPP4) for MERS-CoV [27]; and ACE2 by SARS-CoV-2 [18].

**Table 1:** Human coronavirus species and their receptors.

**Coronavirus species**	**Discovery year**	**Cellular receptor**
HCoV-229E	1966	Human aminopeptidase N (CD13)
HCoV-OC43	1967	9-O-acetylated sialic acid
SARS-CoV	2003	ACE2
HCoV-NL63	2004	ACE2
HCoV-HKU1	2005	9-O-acetylated sialic acid
MERS-CoV	2012	DPP4
SARS-CoV-2	2019	ACE2

### Potential impact of COVID-19 on hemorrhagic stroke in the elderly

At presentation, the most common symptoms in COVID-19 patients are fever, dry cough, and shortness of breath, whereas headache, diarrhea, and vomiting are more rare [3, 28–30]. However, early neurological symptoms (e.g. headache, epilepsy, and unconsciousness), without obvious respiratory symptoms, have been reported for numerous COVID-19 patients [10, 31]. A 2005 case report by Xu et al. provided the first direct evidence that SARS-CoV has the ability to infect the central nervous system (CNS) [32]. A predicted cDNA fragment specific for SARS-CoV was amplified by nested RT-PCR from Vero-E6 cell cultures inoculated with a brain tissue extract from a symptomatic patient, and presence of enveloped virus particles, 80–90 nm in diameter, was found by transmission electronic microscopy [32]. Shortly before this finding, another study had reported the case of a 32-year-old woman with SARS whose cerebrospinal fluid tested positive for SARS-CoV [33]. These findings were further supported by experiments in mice that demonstrated the ability of various CoVs to cause CNS infections [34–36]. Indeed, SARS-CoV-2 shares similar characteristics with SARS-CoV, and both anecdotal and statistical data indicate that neurologic symptoms are not common in COVID-19 patients [10]. Since it is well known that cerebral hemorrhage may result from viral infection of the CNS compromising the neurovascular unit [37–40], available evidence strongly suggest that SARS-CoV-2 infection may greatly increase the incidence of hemorrhagic stroke, especially in at-risk patients.

### Shared risk factors

Hypertension is the most important risk factor for cerebral hemorrhage [41, 42]. Of note, for the 138 COVID-19 confirmed cases analyzed by Wang et al. [30], 43 patients (31.2%) were hypertensive, a proportion that reflects, relative to other diseases, the higher susceptibility to SARS-CoV-2 infection conferred by hypertension. Similar results were recently reported by both Guan et al. [26] and the Novel Coronavirus Pneumonia Emergency Response Epidemiology Team [50]. SARS-CoV-2 infection in humans is mediated by binding of the receptor-binding domain (RBD) of the viral S glycoprotein to ACE2 receptors in host cells, and this in turn may lead to downregulation of ACE2 expression [20, 43]. Since reduced ACE2 expression implies increased Ang II availability, COVID-19 patients with pre-existing hypertension may experience large blood pressure (BP) fluctuations, making them especially susceptible to hemorrhagic stroke episodes.

There is a close relationship between systolic BP variability (SBPV) and poor prognosis of cerebral hemorrhage. Divani et al. reported that elevated SBPV in the first 24 h of admission was related to unfavorable in-hospital prognosis in patients with intracerebral hemorrhage (ICH) [44]. Since BP elevations resulting from downregulation of ACE2 expression may occur after SARS-CoV-2 infection, higher SBPV may be present on admission in hemorrhagic stroke patients affected by COVID-19. Therefore, the management of BP might require additional attention during the hyper-acute and acute hemorrhagic stroke phases in COVID-19 patients, as both high absolute BP levels and high BP fluctuations are main determinants of cerebral hemorrhage prognosis.

Diabetes is also an independent risk factor for hemorrhagic stroke [42]. Huang et al. reported that among 41 patients with laboratory- confirmed SARS-CoV-2 infection, 8 (20%) cases had diabetes; this again represents a higher proportion of comorbidity cases compared with other diseases [45]. Indeed, available data suggest that among COVID-19-confirmed cases with underlying chronic diseases, diabetes ranks second after hypertension [29, 45].

Elevated plasma D-dimer levels were associated with increased risk of hemorrhagic stroke [41]. Recently, Chen et al. conducted a retrospective, single-center study including 99 patients with COVID-19 and found elevated D-dimer levels in 36 patients (36%) [28]; however, mortality rate for this subgroup was not reported. Meanwhile, in a similar study assessing 191 COVID-19-confirmed patients, D-dimer greater than 1 mg/L on admission was associated with significantly increased odds (p = 0.0033) of in-hospital death [46]. Of note, a recently posted pre-print article reporting on 248 consecutive COVID-19 cases in Wuhan found D-dimer elevation (≥ 0.50 mg/L) in 74.6% (185/248) of the patients. D-dimer levels correlated with disease severity, and values >2.14 mg/L predicted in-hospital mortality with a sensitivity of 88.2% and specificity of 71.3% [47].

Surprisingly, two recent studies have reported an association between SARS-CoV-2 infection and the incidence of stroke [31, 48]. A single center, retrospective, observational study by Li et al reported a 5% risk of ischemic stroke and a 0.5% risk of cerebral hemorrhage in 221 patients with SARS-CoV-2 infection from Wuhan, China [48]. In this cohort, patients with new onset stroke are obviously older, more likely to present with severe COVID-19 and have the above risk factors including hypertension, diabetes and elevated plasma D-dimer levels [48]. Another study of 214 patients reported 5 (5.7%) developed acute cerebrovascular diseases including 4 (4.6%) patients with ischemic stroke and 1 (1.1%) with cerebral hemorrhage in severe patients with COVID-19 [31]. Nevertheless, further studies including larger sample sizes, more exhaustive assessment of patients’ clinical histories, and additional molecular analysis are clearly needed to determine in which cases stroke is directly triggered by SARS-CoV-2 infection, or it occurs coincidentally [49].

### Convergence of inflammatory mediators

Inflammatory monocyte-macrophages (IMMs) and neutrophils are major sources of cytokines and chemokines involved in the pathogenicity of SARS-CoV-2 [50]. Some of these factors represent classical inflammatory biomarkers associated with secondary brain injury following cerebral hemorrhage and may have prognostic value in hemorrhagic stroke patients [51–55]. Lattanzi et al. recently reviewed available evidence pointing to the relevance of assessing the neutrophil-to-lymphocyte ratio (NLR) to determine inflammatory status in ICH patients [54]. In turn, newer studies confirmed NLR’s predictive value for prognosis of ICH [56, 57]. Neutrophil-derived matrix metalloproteinases (MMPs) are upregulated after acute ICH, contributing significantly to tissue destruction and activation of neuro-inflammatory cascades [54]. Accordingly, research suggests that it may be possible to mitigate brain damage by early, short-term inhibition of MMPs [53]. Napoli et al. reported that increased concentrations of serum C-reactive protein (CRP), a marker of inflammation, may be an independent predictor of ICH outcome [52]. Nevertheless, it should be considered that interethnic genomic differences may influence CRP status and its predictive values on different stroke phenotypes. Another marker, namely serum neutrophil gelatinase-associated lipocalin (NGAL), a member of the lipocalin family of proteins associated with transport of small hydrophobic molecules, plays an important role in the innate immune response and has also been identified as an independent predictor for outcome following hemorrhagic stroke [51]. Given that these inflammatory biomarkers have been associated with both SARS-CoV-2-related cytopathic effects and hemorrhagic stroke outcome, it would be worthwhile to explore which changes in inflammatory biomarkers occur after hemorrhagic stroke and their predictive value in patients with and without COVID-19. This would allow to better define reliable indices of hemorrhagic stroke severity and functional recovery.

Substantially reduced peripheral lymphocyte counts were evident in severe COVID-19 cases [28–30, 45, 58]. Xu et al. reported pathological findings of lung, liver, and heart biopsies, as well as blood cell analysis, from a patient who died of COVID-19 [59]. The findings showed infiltration of IMMs in the lung, whereas peripheral CD4 and CD8 T cells were reduced in number but overactivated. The authors suggested that severe immune injury in this patient was due to overactivation of T cells, manifested by increased representation of highly pro-inflammatory CCR6+ Th17 CD4 T cell subsets and enhanced cytotoxic capacity of CD8 T cells. These data suggest that although lymphopenia is a common feature in patients with COVID-19, it may be paralleled by a pro-inflammatory phenotypic switching in T cell subsets that could be critically associated with disease severity and mortality [9, 59].

In addition, it was suggested that like SARS-CoV, SARS-CoV-2 also acts on lymphocytes in the respiratory mucosa, leading to a systemic “cytokine storm” concomitant with reduced peripheral blood lymphocytes which impairs cellular immune function [28]. This effect will be clearly potentiated by immune senescence, a well-described phenomenon in many middle-aged and elderly people [60], and aggravated by underlying conditions such as hypertension, diabetes, and cerebrovascular disease. This evidence points to worsened outcomes for patients with COVID-19 and cerebral hemorrhage comorbidity.

### Possible mechanisms underlying COVID-19 effects on hemorrhagic stroke in the elderly

### ACE2 expression

Soon after the COVID-19 outbreak, investigations confirmed that the ACE2 receptor, abundantly expressed in lung alveolar epithelial cells, enables SARS-CoV-2 entry into host cells through the RBD of the virus’ S glycoprotein [12, 61, 62]. The RBD that confers ACE2 binding specificity is part of the S1 subunit of the large ectodomain of the S protein. The ectodomain contains also an S2 subunit, which mediates fusion between the viral and host cell membranes [61]. A ternary structure of the RBD of SARS-CoV-2 was obtained by molecular simulation, revealing that the structure is essentially superimposable (72% identity) to that of SARS-CoV, except for a flexible loop with CNGVEGFNC that replaces the rigid loop with CTPPALNC present in SARS-CoV [61]. Further analysis indicated that the unique F486 residue in the flexible loop can penetrate deep into a hydrophobic pocket in ACE2 formed by F28, L79, Y83, and L97 [61].

ACE2 was identified in 2000 as a homolog of the angiotensin-converting enzyme (ACE), although with different substrate specificity [63]. ACE2 primarily acts on angiotensin II (Ang-II), a major bioactive peptide [43], to generate the vasodilatory heptapeptide Ang-(1-7), while ACE acts on angiotensin I (Ang-I) to generate Ang-II [43]. ACE2 counterbalances the vasopressor effect of the ACE/Ang-II/AT1 axis by stimulating vasodilation through the ACE2/Ang-(1-7)/MasR axis [64, 65]. Demonstrating the adversarial relationship between ACE and ACE2, Crackower et al. reported that heart function is impaired in *ace2*-deficient mice, and this effect can be rescued by ablation of ACE expression [66]. ACE2 expression is widely distributed across different cells and tissues. To date, it was identified in epithelial cells of the oral mucosa [62], pulmonary alveolar type II cells [67–69], esophagus upper and stratified epithelial cells, absorptive enterocytes from ileum and colon [69], cholangiocytes [70], myocardial cells, kidney proximal tubule cells, and bladder urothelial cells [46]. In addition, ACE2 expression has also been detected in vascular endothelial and smooth muscle cells [71] and in some neurons [43, 64, 71–73], including those in the cardio-respiratory center of the brainstem [43]. The widespread expression of ACE2 is thus consistent with the reported effects of SARS-CoV-2 on multiple tissues and organs. Binding of SARS-CoV-2 to ACE2 receptors in brain blood vessels may trigger the release of proinflammatory cytokines and chemokines such as interleukin-6 (IL-6) and tumor necrosis factor (TNF), leading to activation and extravasation of lymphocyte subsets, neutrophils, and macrophages with subsequent neurological manifestations [74]. On the other hand, neuronal ACE2 expression could also be a significant factor in COVID-19 cases associated with cerebral hemorrhage. Research on the 2003 SARS outbreak concluded that downregulation of ACE2 expression occurred in infected organs, including lungs [75], kidney [43], heart [76], liver [43], and brain [43]. Similarly, a study by Chen et al. reported decreased ACE2 expression in the lungs of COVID-19 patients [61].

**Figure 1:**
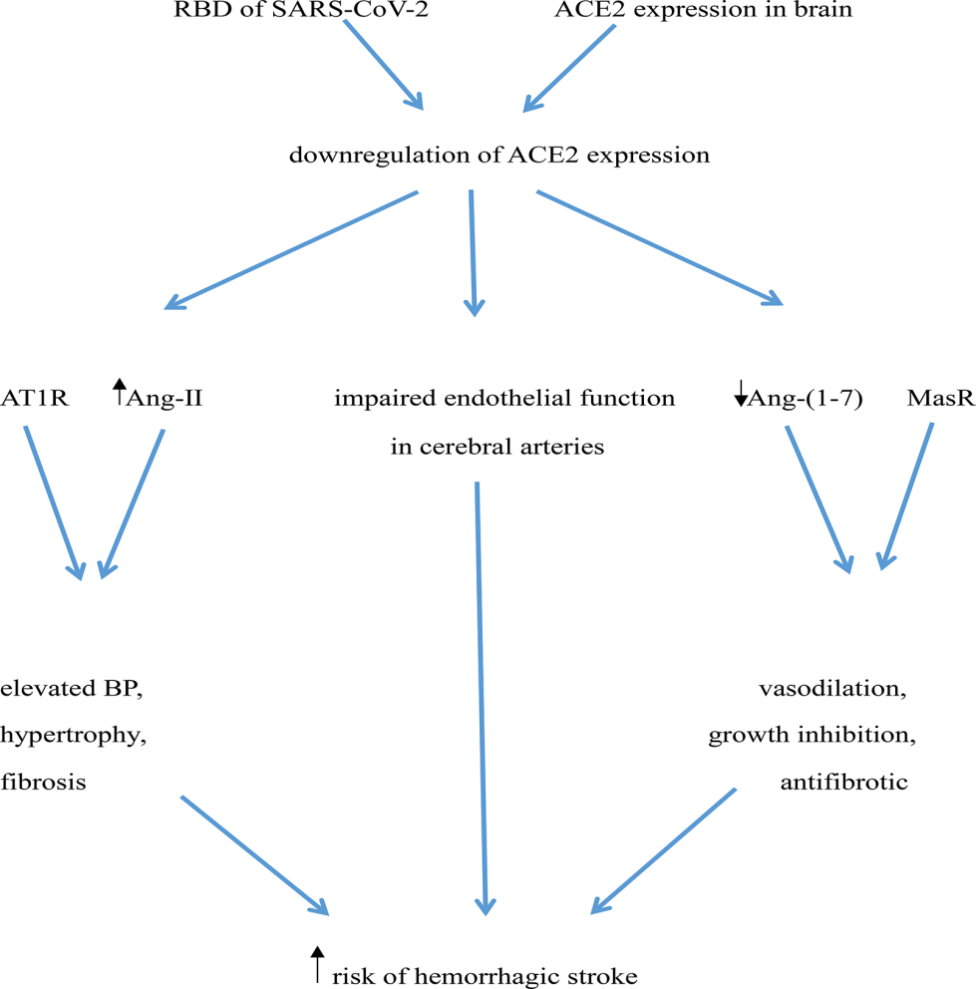
**Potential mechanisms mediating increased risk of hemorrhagic stroke in COVID-19 patients.** The RBD of SARS-CoV-2’ spike protein interacts with ACE2, leading to ACE2 downregulation. ACE2 deficiency impairs endothelial function in cerebral arteries and determines an increase in Ang-II levels, which elevates BP through activation of AT1 receptors (AT1R). Simultaneously, reduced ACE2 leads to a decrease in Ang (1-7) levels, weakening its vasculo-protective effects mediated by Mas receptor (MasR) activation.

Downregulation of ACE2 expression may increase risk of hemorrhagic stroke in several ways: i) ACE2 deficiency in the brain may impair endothelial function in cerebral arteries, leading to a 4-fold elevation in the risk of cerebrovascular events, including hemorrhagic stroke [77]; ii) Downregulation of ACE2 expression may increase local Ang-II levels, which acting on AT1 receptors may rise BP and facilitate hypertrophy and fibrosis [64]; iii) Decreased ACE2 expression would also lead to reduced generation of Ang (1-7) and depression of Ang (1-7)/MasR signaling, thus preventing its vasodilatory, growth inhibiting, and antifibrotic actions [64, 78] (Figure 1).

It calls for special attention the fact that COVID-19 may exacerbate any underlying hypertension and put patients at higher risk for hemorrhagic stroke. Several mechanisms may contribute to hemorrhagic stroke in hypertensive patients infected with SARS-CoV-2. These include fibrinoid necrosis, promoted by increased vascular pressure [79], and extensive structural and functional alterations in endothelium and smooth muscle in intracerebral arteries, often aggravated by atherosclerosis, especially in the elderly [80].

### Endothelial dysfunction at the blood-brain barrier

The BBB is a semi-permeable structure consisting of a well-defined basement membrane and endothelial cells bound by tight junctions that limit the passage of macromolecules into the brain parenchyma. The BBB lies in close apposition to brain cell types, including pericytes, astrocytes, microglia, and neurons, and is especially susceptible to damage by both hypertension and diabetes [81, 82]. Xu et al. reported that a chemokine, i.e. the monokine/Mig/CXCL9, induced by IFN-g mostly in glial cells, might be involved in the brain immunopathology triggered by SARS [32]. Elevated Mig levels in the blood are correlated with brain infiltration of CD68^+^ monocytes/macrophages and CD3^+^ T lymphocytes in the brain [32]. Given the similarities between SARS-CoV-2 and SARS-CoV, this mechanism deserves further exploration as it may lead to therapeutic strategies to prevent or attenuate brain pathology in COVID-19 patients.

The BBB is a dynamic and complex structure that helps maintain brain homeostasis and compensates fluctuations in the systemic circulation [83]. Expression of ACE2 in endothelial cells of the BBB may be a gateway for SARS-CoV-2 entry into the brain [83]. Moreover, the ensuing ACE2 down-regulation, compounded by age-related ACE2 deficiency in older patients, might further increase endothelial dysfunction and risk of ICH [77]. More studies are needed to ascertain the impact of ACE2 expression at the BBB and its effect on SARS-CoV-2-mediated CNS symptoms, particularly ICH.

### Immunity and inflammation

There is accruing evidence that viral CNS infections may cause hemorrhage stroke [37, 39, 84]. The pathogenesis may involve cytokine, chemokine, and protease actions increasing BBB permeability, and damage and/or demise of the neurovascular unit during the necrotizing process [37]. Although the specific mechanisms remain unclear, it is obvious that the type and extent of the immune response triggered by the SARS-CoV-2 determine symptoms severity. A recent study from Anderson et al. revealed that bats, the most likely source of the novel SARS-CoV-2, have evolved a highly specific innate immune response characterized by a large expansion of the type I interferon gene family [85]. While this may clarify the basis of bats’ immune resistance to SARS-CoV-2, there are still many open questions about the mechanism(s) mediating immune defense against CoV-2 in humans. In this regard, it will be very valuable to ascertain and compare immunological (i.e. T cell status, cytokine expression) and genetic (i.e. HLA haplotypes) profiles between symptomatic and asymptomatic COVID-19 patients, which have shown to influence responses to recent viral outbreaks [86]. This should allow predicting why high viral replication early in the course of infection would lead to the “cytokine storm” characteristic of severe COVID-19 cases [50].

### Anxiety and stress

The current COVID-19 outbreak has undoubtedly increased anxiety, fear, and stress in many people around the world. Social stress, anxiety, and depression are potential risk factors for hemorrhagic stroke, therefore adequate management of these conditions is a key aspect in primary prevention of cerebrovascular disease [87, 88]. The locus coeruleus, a structure in the brainstem, consists mainly of adrenergic neurons that play a crucial role in the genesis of anxiety by releasing catecholamines that critically influence the stress response [89]. Indeed, research has shown that excessive adrenergic stimulation by catecholamines could lead to severe vasospasm and microcirculation disturbances, thus increasing the risk of hemorrhagic stroke [90].

### Aging

Although people of all ages can be infected, middle-aged and elderly people are most severely affected by COVID-19, suggesting that aging is a prominent risk factor. Accordingly, it seems logical that the risk of hemorrhagic stroke in COVID-19 patients would increase significantly with age, although a recent article by Oxley et al reported COVID-19-related stroke episodes occurred in five young patients [91]. Based on available evidence, Camacho et al. concluded that age is a strong risk factor for hemorrhagic stroke, the deadliest stroke type [92]. Their study highlights several age-related processes and pathologies, including cerebral microembolism, white matter lesions, vascular basement membrane thickening, and increased BBB permeability, which determine endothelial damage, changes in vessel elasticity, and ensuing fluctuations in blood flow and pressure that cause loss of autoregulation and increase the risk of ICH [92].

Research on both animal models and humans indicated that aging is closely associated with endothelial dysfunction and oxidative stress in cerebral arteries [93–97]. Moreover, studies in rodents suggested that these deleterious effects can be promoted by alterations in the RAS system in aged brains. Specifically, works by Pena-Silva et al. [77] and Labandeira-Garcia et al. [98] suggested that age-related downregulation of ACE2 and AT2 expression may promote vascular dysfunction because the anti-inflammatory/anti-oxidant effects of AngII/AT2 and Ang1-7/MasR signaling are overridden by pro-inflammatory/pro-oxidant signaling through the AngII/AT1 axis. Although confirmatory data in humans is still needed, these studies provide strong support for the overall concept that brain RAS activity has a critical effect on cerebrovascular function during aging and may contribute to endothelial dysfunction, oxidative stress, and risk of hemorrhagic stroke.

## CONCLUSIONS

COVID-19 emerged as a new human infectious disease caused by SARS-CoV-2, a novel coronavirus. A significant proportion of COVID-19 cases, especially older patients, manifest neurological, rather than respiratory, symptoms on admission and may be at higher risk of developing cerebral hemorrhage. The mechanisms by which COVID-19 may promote hemorrhagic stroke in the elderly are not yet clear, but may involve downregulation of ACE2 expression secondary to SARS-CoV-2 binding to neurovascular ACE2 receptors. This might increase Ang-II expression and decrease Ang (1-7) expression, leading to severe BP elevation. increased BBB permeability, and extensive alterations in endothelium and smooth muscle function in intracerebral arteries. The patients most gravelly affected by COVID-19 have underlying hypertension disease, which greatly increases the risk of hemorrhagic stroke. Since SBPV in the first 24 h of admission predicts cerebral hemorrhage outcome, special attention should be paid to management of BP in at-risk COVID-19 patients. Predisposing factors may be compounded in COVID-19 patients by the inability of their immune system to efficiently prevent or counteract the pernicious effects of the pro-inflammatory cytokines released upon infection. In addition, anxiety and stress may lead to enhancement of adrenergic tone and trigger vasospasm and microcirculation disturbances, further contributing to cerebrovascular symptoms. In light of this, exploring the changes in inflammatory biomarkers occurring in COVID-19 patients with CNS symptoms suggestive of incipient stroke would aid diagnosis and treatment to avoid irreversible outcomes.
